# LINAC stereotactic radiosurgery for trigeminal neuralgia –retrospective two-institutional examination of treatment outcomes

**DOI:** 10.1186/s13014-018-1102-2

**Published:** 2018-08-22

**Authors:** Ali Rashid, Bogdan Pintea, Thomas M. Kinfe, Gunnar Surber, Klaus Hamm, Jan P. Boström

**Affiliations:** 1 Department of Radiosurgery and Stereotactic Radiotherapy, MediClin Robert Janker Clinic and MediClin MVZ Bonn, Villenstrasse 8, 53129 Bonn, Germany; 2 Department of Neurosurgery, BG University Hospital Bergmannsheil, Bürkle-de-la-Camp-Platz 1, 44789 Bochum, Germany; 3 Department of Psychiatry, Division of Medical Psychology, University Hospital of Bonn, Sigmund-Freud-Strasse 25, 53105 Bonn, Germany; 4 CyberKnife Zentrum Mitteldeutschland, Nordhäuser Strasse 74, 99089 Erfurt, Germany; 5 Department of Neurosurgery, University of Bonn Medical Center, Sigmund-Freud-Str. 25, 53105 Bonn, Germany

**Keywords:** Radiosurgery, Linear accelerator, Trigeminal neuralgia, Outcome

## Abstract

**Background:**

In this pooled 2-center series LINAC radiosurgery (SRS) has been applied as a treatment option for a subset of refractory trigeminal neuralgia (TN) patients. This study approached to retrospectively assess the efficacy and safety of LINAC SRS and to provide a brief overview addressed to the technical development from frame-based towards frameless robotic SRS.

**Methods:**

From 2001 to 2017 *n* = 55 patients (pts) were treated, *n* = 28 were female (51%), mean age: 66 years (range 36–93 years); TN etiology: 37 classic TN, 15 multiple sclerosis (MS)-related TN, 2 symptomatic TN, and 1 atypical TN. Previous treatment was present in *n* = 35 (63.6%) pts. (some multiple or combined) with *n* = 23 microsurgical vascular decompression and *n* = 17 percutaneous retrogasserian rhizotomy. A 6 MV LINAC (4–5 mm collimators) was applied in all pts. (*n* = 26 framebased - *n* = 29 frameless robotic). The dorsal root entry zone (DREZ) was targeted in n = 35 cases and the retrogasserian target in *n* = 20 pts. with a homogeneous dose for the entire study cohort (90 Gy). SRS outcome was measured using the Barrow Neurological Institute (BNI) score for pain and hypaesthesia and statistically evaluated by univariate and multivariate analyzes.

**Results:**

Mean follow-up (FU) was 30 months (2 lost FU); the total rate of post SRS BNI pain I-IIIa (=painfree w or w/o medication) was 69% (88% for the classic TN pts), 29% (38.8% classic TN) were classified as BNI pain I-II (=painfree w/o medication). A BNI hypaesthesia II-III was present in 9.4% (*n* = 5) and BNI hypaesthesia IV in *n* = 2. Between groups analysis demonstrated no correlation of SRS responsiveness with age, gender, MS- or not MS-associated TN, previous surgery, framebased/frameless robotic SRS. DREZ targeting significantly better suppressed TN compared to RG targeting (*p* = 0.01). Additionally, a statistical trend for a better BNI pain outcome (*p* = 0.07) along with a significant increase in BNI hypaesthesia (*p* = 0.01) was found when using a larger partial trigeminal 70 Gy volume.

**Conclusion:**

Our retrospective analysis support LINAC SRS as an effective and safe treatment option in TN. Frameless robotic SRS of TN is safe when using a dedicated LINAC system. A target definition closer to the brainstem and tendencially a larger target volume were associated with a better outcome for pain.

## Background

Trigeminal neuralgia (TN) represents a chronic, debilitating pain condition of the trigeminal nerve and corresponding branches with an incidence of around 12 per 100.000, disproportionately affecting elderly and female patients [1]. According to the International Classification of Headache Disorders (The International Headache Society (IHS) Criteria, 3rd edition 2014) two subtypes are distinguished. The first, the classic form of TN (with no apparent cause other than neurovascular compression) is characterized by sudden shocks of severe facial pain lasting from a fraction of a second to 120 s in the distribution of the facial divisions of the trigeminal nerve often precipitated by innocuous stimuli to the affected side of the face. The second subtype, the non classic form constitute of different trigeminal neuropathies attributed to other apparent causes than a neurovascular conflict like multiple sclerosis or space occupying lesions of the cerebello-pontine angle [2].

First-line treatment in the pain management of TN remains pharmacotherapy (carbamazepine, gabapentin, and oxcarbazepine) [1]. In case such medications are found to be inappropriate effective and/or associated with adverse side effects, invasive approaches, namely microsurgical vascular decompression (MVD) or ablative procedures like rhizotomy may be utilized [3–5]. Surgical invasive MVD (“Jannetta” decompression) requires an open craniotomy and may be associated with surgical-related complications including facial numbness, facial palsy, CSF leaks, hearing deficits, and incisional infections. Nevertheless, previous studies indicate that MVD provides long-lasting pain relief with a responder rate of around 80% [6–8].

Alternatively, minimal-invasive percutaneous retrogasserian rhizotomies (PRR) applying glycerol, radiofrequency, or ballon compression have been found to be act effectively in providing initial pain relief. Adverse events occurred more frequently than in MVD with the majority being sensory issues [3, 9, 10]. Of note, in a minor proportion severe sensory deficits (anaesthesia dolorosa) were recorded depending on the applied technique. However, PRR studies demonstrated limited sustained TN pain suppression [9, 11].

Non-invasive stereotactic radiosurgery (SRS) has been reported to be a viable treatment option for TN since firstly introduced by Leksell [12]. SRS represents a non-invasive and safe alternative to minimal-invasive PRR or invasive, surgical MVD after failure of pharmacological and behavioural pain interventions [13, 14].

A rising number of clinical data has been published with an appropriate observation period with respect to Gamma Knife® (GK) SRS for the treatment of TN [15–17]. In a large-scale review including 8 GK series with 1215 TN patients, Sheehan et al. observed a success rate of > 75% and a pain free rate of 50% along with a complication rate of 13% and a TN recurrence rate of 20% assessed over 20 months post GK SRS [18]. However, linear accelerator (LINAC) and robotic-assisted SRS has been increasingly applied recently for TN treatment [19–24]. Varela-Lema et al. reviewed 11 LINAC cohort studies, of which 3 were conducted prospectively, enrolling data of 549 patients. Overall, a comparable success rate of 75–95% with a 1 y recurrence rate of 5–29%, but a more variable rate of mild-to-moderate adverse events (mainly hypaesthesia) occurring in 7.5–52% of LINAC SRS treated patients was noted [25]. Hence, systematic multicenter-based studies addressed to TN responsiveness and possible associations with dose-response interaction on target volume, target point, age, gender, adverse events, and TN origin using LINAC-based SRS have to address remaining clinically relevant open questions.

We here report on a retrospective assay of a pooled bi-centric LINAC SRS study of TN treatment covering an observation period of 15 years and assessed TN outcome, adverse events, and TN origin (classic versus non-classic). We compare the treatment results for the two most used SRS targets, the dorsal root entry zone (DREZ) and the retrogasserian (RG) target. We have additionally determined the 70 Gy volume (almost spherical), which seems empirically the minimal effective dose, and evaluated volumetrically the proportion of the trigeminal nerve within this 70 Gy volume. We have correlated these volumes with outcome as regards the therapy success as well as the side effect profile. In addition, we briefly describe the technical development from frame-based to frameless robot-assisted and image-controlled SRS with an identical LINAC irradiation system.

## Methods

### Study cohort

This is a two-centre retrospective study done at the “CyberKnife-Centrum Mitteldeutschland” in Erfurt, Germany and at the “MediClin Robert Janker Klinik” in Bonn, Germany. From 2001 through 2017, 55 patients (28 female, mean age 66 years, range 36 to 93 years) with clinically diagnosed TN were treated at our institutions and listed in a prospective clinical database (see Table 1). For all patients informed consent was obtained in accordance with the tenets of the Declaration of Helsinki, because of the retrospective nature of the study no special approval by the Ethics Committee was necessary.

**Table 1:** Descriptive statistics

No	Sex	Age	TN etiology	Carbamazepin mono/combined = 1, other = 0	Pretreatment	Side	Target RG / DREZ	Collimation	Localisation framebased / robotic	Dose (Gy)	70 Gy TN vol. (cc)	FU (mo)	BNI hypaesthesia	BNI pain last FU
1	f	81	classic	1	MVD	right	DREZ	4 mm Cones	framebased	90	–	116	I	I
2	m	78	classic	1	2× MVD	left	DREZ	4 mm Cones	framebased	90	0,013	62	II	I
3	m	74	classic	1	MVD	left	DREZ	4 mm Cones	framebased	90	0,014	28	I	IIIa
4	m	44	classic	1	MVD	right	DREZ	4 mm Cones	framebased	90	0,012	200	II	I
5	m	93	classic	1	MVD	right	DREZ	4 mm Cones	framebased	90	0,011	9	I	I
6	f	83	classic	1	MVD, PRR	left	DREZ	4 mm Cones	framebased	90	0,015	14	I	IIIa
7	m	82	classic	0	–	left	DREZ	4 mm Cones	framebased	90	0,009	83	II-III	IIIa
8	m	70	classic	0	MVD, PRR	right	DREZ	4 mm Cones	framebased	90	0,02	107	II-III	IIIa
9	f	63	classic	1	–	left	DREZ	4 mm Cones	framebased	90	0,007	104	I	IIIa
10	f	72	classic	1	MVD	right	DREZ	4 mm Cones	framebased	90	0,009	102	I	IIIa
11	m	75	classic	1	–	left	DREZ	4 mm Cones	framebased	90	0,006	–	–	–
12	m	51	MS	0	MVD	left	DREZ	4 mm Cones	framebased	90	0,03	24	II	IIIb
13	f	36	MS	1	PRR	left	DREZ	4 mm Cones	framebased	90	0,013	90	I	I
14	m	76	classic	1	–	right	DREZ	4 mm Cones	framebased	90	0,012	2	I	IIIa
15	f	55	classic	0	MVD	left	DREZ	4 mm Cones	framebased	90	0,014	14	I	IIIa
16	f	63	MS	1	4× PRR	left	DREZ	4 mm Cones	framebased	90	0,006	67	I	IIIa
17	m	78	classic	1	–	right	DREZ	4 mm Cones	framebased	90	0,048	2	I	IIIa
18	m	70	classic	0	MVD, PRR	left	DREZ	4 mm Cones	framebased	90	0,021	10	I	IIIa
19	f	69	MS	1	–	right	DREZ	4 mm Cones	framebased	90	0,016	27	I	IIIa
20	f	74	classic	1	MVD, 2× PRR	right	DREZ	4 mm Cones	framebased	90	0,032	22	I	IIIa
21	f	74	classic	0	RT	right	DREZ	4 mm Cones	framebased	90	0,02	19	I	I
22	f	58	MS	0	–	left	DREZ	4 mm Cones	framebased	90	0,039	34	II-III	I
23	m	47	MS	1	–	left	DREZ	4 mm Cones	framebased	90	0,033	–	–	–
24	f	46	MS	1	–	left	DREZ	4 mm Cones	framebased	90	0,044	22	I	IIIa
25	f	67	classic	0	–	right	DREZ	4 mm Cones	framebased	90	0,021	22	II-III	I
26	f	76	classic	0	PRR	left	DREZ	4 mm Cones	framebased	90	0,04	20	II-III	I
27	m	51	sympt.	1	PRR	left	RG	4 mm μMLC	robotic	90	0,013	69	II	V
28	m	71	classic	0	MVD, 2× PRR	right	DREZ	5 mm Cones	robotic	90	0,04	7	I	I
29	f	60	classic	1	PRR	right	RG	4 mm μMLC	robotic	90	0,023	2	II	II
30	m	64	classic	1	MVD	right	RG	4 mm μMLC	robotic	90	0,033	12	I	V
31	m	75	classic	1	MVD	left	RG	4 mm μMLC	robotic	90	0,048	22	II	IIIa
32	f	83	classic	1	–	right	RG	4 mm μMLC	robotic	90	0,047	9	II	IIIa
33	f	44	MS	0	5× PRR	right	RG	4 mm μMLC	robotic	90	0,021	10	I	IIIa
34	f	59	MS	0	MVD, PRR	left	RG	4 mm μMLC	robotic	90	0,03	8	I	IIIb
35	f	56	MS	0	–	left	DREZ	5 mm Cones	robotic	90	0,024	37	I	IIIb
36	f	80	classic	–	–	left	DREZ	5 mm Cones	robotic	90	0,035	42	II	IIIb
37	m	43	MS	1	2× PRR	right	RG	4 mm μMLC	robotic	90	0,05	30	I	IIIb
38	f	76	classic	0	–	left	RG	4 mm μMLC	robotic	90	0,042	18	IV	I
39	f	84	classic	1	PRR	left	RG	4 mm Cones	robotic	90	0,008	11	I	IIIa
40	f	79	classic	0	–	right	RG	4 mm Cones	robotic	90	0,008	12	I	I
41	f	51	MS	1	–	right	RG	4 mm Cones	robotic	90	0,007	4	I	V
42	m	51	MS	1	PRR	_,_right	RG	4 mm Cones	robotic	90	0,004	2	II	V
43	m	44	MS	1	PRR	right	RG	4 mm Cones	robotic	90	0,004	6	II	IIIa
44	m	66	sympt.	0	RT	left	RG	4 mm Cones	robotic	70	0,001	7	I	V
45	m	69	MS	1	–	right	RG	4 mm Cones	robotic	90	0,007	12	I	V
46	m	57	classic	1	2× MVD, PRR	right	DREZ	5 mm Cones	robotic	90	0,042	17	I	IIIa
47	f	40	classic	1	2× MVD	right	DREZ	5 mm Cones	robotic	90	0,036	23	III	V
48	m	76	classic	1	2× MVD	right	DREZ	5 mm Cones	robotic	90	0,034	18	II	IIIa
49	m	64	classic	1	2× MVD	right	DREZ	5 mm Cones	robotic	90	0,031	21	I	I
50	m	78	classic	0	–	left	RG	4 mm Cones	robotic	90	0,008	14	I	IIIa
51	f	73	classic	1	–	left	RG	4 mm Cones	robotic	90	0,007	5	II	II
52	f	78	atyp.	1	MVD (left)	right	RG	4 mm Cones	robotic	90	0,008	8	IV	IV
53	f	74	classic	1	–	right	RG	4 mm Cones	robotic	90	0,005	6	I	II
54	f	81	classic	1	MVD	left	DREZ	5 mm Cones	robotic	90	0,039	7	I	IV
55	f	64	classic	1	2× MVD	right	DREZ	5 mm Cones	robotic	90	0,049	6	II	I

*MS* multiple sclerosis, *MVD* microsurgical vascular decompression, *PRR* percutaneous retrogasserian rhizotomy, *RT* radiotherapy, *DREZ* dorsal root entry zone, *RG* retrogasserian, *BNI* Barrow Neurological Institute, *FU* follow-up, *m* male, *f* female, *mo* month, *vol.* volume, *sympt.* symptomatic, *atyp.* atypical

The TN was classified in accordance with the international classification of headache disorders of the international headache society [2]. *N* = 37 (67.3%) patients (pts) suffered the classic (=type 1) form, the rest were pts. with non classic (=type 2) trigeminal neuropathies attributed to other apparent causes than a neurovascular conflict like multiple sclerosis (MS, 15 pts./27.3%) or space occupying lesions (2 symptomatic cases/3.6%), and other (1 atypical TN/1.8%).

Mean age of patients with non classic TN (> MS related) was significantly lower (55 vs. 73 years, *p* = 0.0001).

From these 55 patients 35 pts. (63.6%) were surgically pretreated regarding the TN, 23 pts. (41.8%) were pretreated with MVD and 17 pts. (30.9%) with PRR.

All pts. had already been adjusted by medication with (Ox-) Carbamazepin (37/55), Pregabalin (17/55), Gabapentin (13/55), opiate/opioide analgetics (14/55) and/or others.

### Stereotactic radiation treatment and FU-assessment

The majority of pts. (*n* = 46) underwent single-session stereotactic radiosurgery (SRS) using the Novalis®-system (Brainlab® AG, München, Germany), the rest of the patients (*n* = 9) were treated with the Cyberknife®-system (Accuray® Inc., Sunnyvale, California, USA).

In the years 2001 to 2012 the framebased Novalis®-system was used (*n* = 26), after that only frameless radiosurgery was performed, using the Novalis®-system (*n* = 20) and the Cyberknife®-system (*n* = 9). The basic techniques have already been described elsewhere [21–24, 26–28].

In all cases a 6 MV LINAC with 4–5 mm collimators (cones/micro multi-leaf collimator (μMLC)) was used, SRS was *n* = 26 framebased and *n* = 29 frameless robotic.

Patients undergoing first-time SRS treatments typically received a peak dose (dose to 100% isodose point) of 90–95 Gy delivered in 9 to 16 non coplanar arcs (Novalis®-system, Brainlab® AG, München, Germany) or approx. 160 beams (Cyberknife®-system, Accuray Inc., Sunnivale, USA). In all but one cases the prescription dose was 90 Gy. This single patient was pretreated elsewhere with conventional radiotherapy because of cancer.

The 20% isodose line at the limit of the brainstem surface was used as constraint for the brainstem.

The stereotactic CT was used for the bony information, in particular for depicting the transition of the trigeminal nerve from intracranial space to the cavum Meckeli. The 3 D T1 sequence with contrast enhancement served for the contouring of the brainstem. For the identification of the trigeminal nerve we routinely used fast gradient echo steady-state sequences (Fig. 2).

**Fig. 1:**
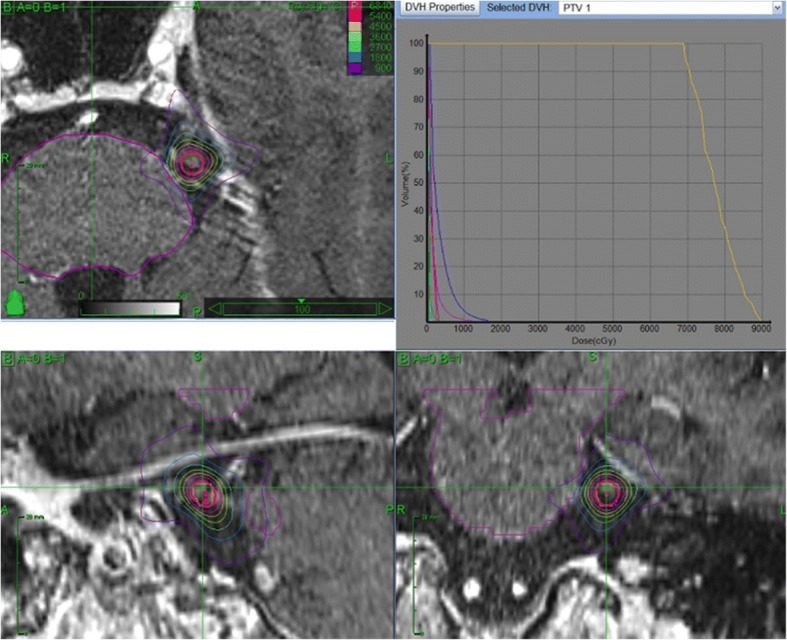
Dorsal root entry zone (DREZ) target. Typical plan with DREZ target (red circle), isodoses and dose-volume histograms (DVH) for trigeminal nerve (yellow line) and for different organs at risk (OAR) (blue line = brainstem)

**Fig. 2:**
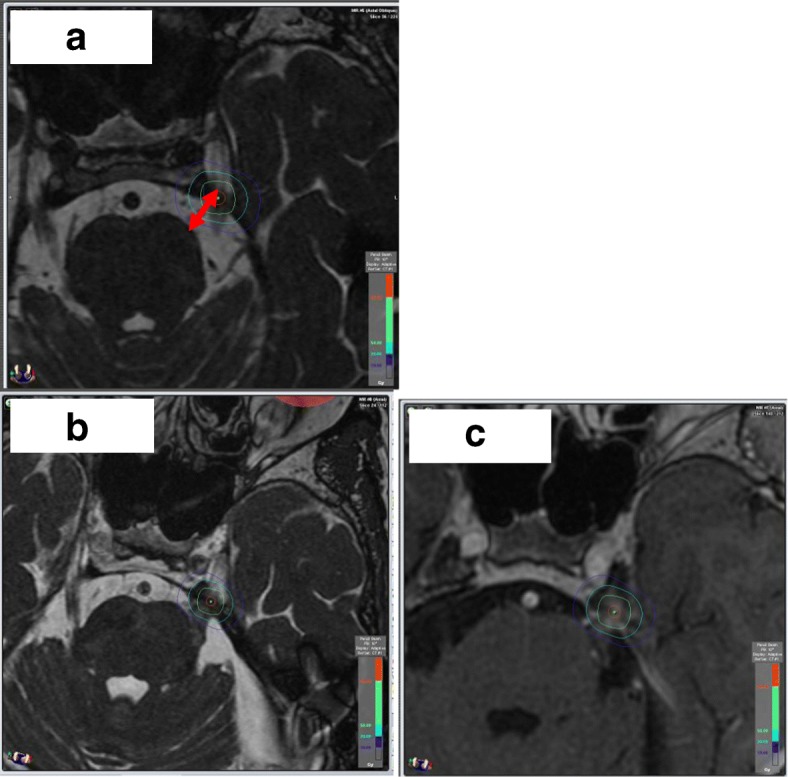
**a**-**c** Retrogasserian (RG) target post Janetta decompression. Different situation pre (**a**) and post (**b**) Janetta decompression with narrowed space in the prepontine cistern (red arrow). Typical small dot-shaped contrast enhancement 2 months after radiosurgery (**c**)

In *n* = 35 cases the target point was the dorsal root entry zone (DREZ) (Fig. 1) and in *n* = 20 cases retrogasserian (RG) (Fig. 2). After intervention, the patients received a strict follow-up (FU) care (after 2 months, 6 months, yearly) consisting of a clinical and radiological investigation with MRI (minimum T1 with gadolinium and steady state sequences). The FU MRI results were merged and volumetrically assessed using the Brainlab® iPlan® RT Image version 4.1.2 or the BrainSCAN® version 5.31 (both Brainlab® AG, München, Germany), for examples see Figs. 1 and 2.

**Fig. 3:**
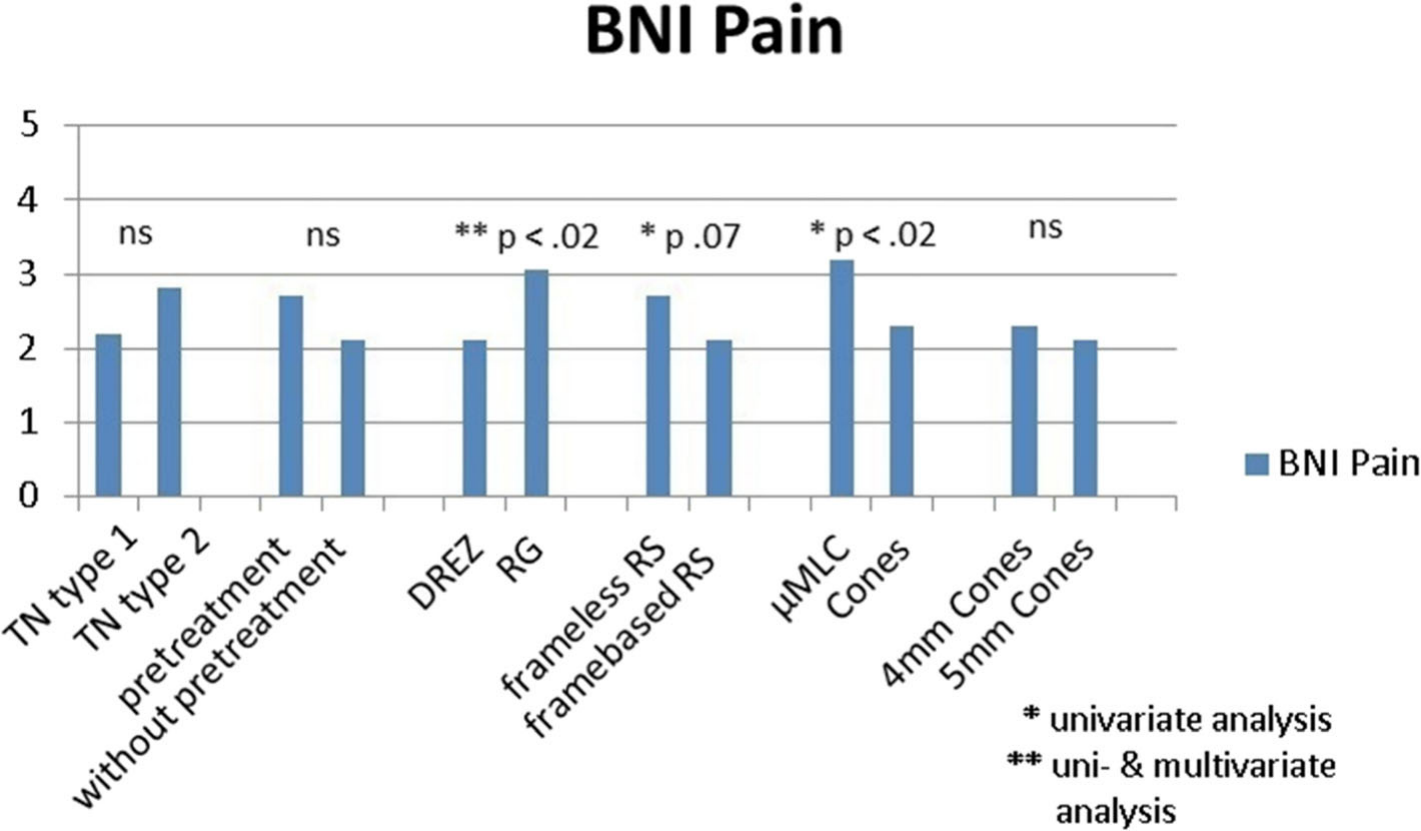
BNI pain scores for different subgroups. Results of uni- and multivariate analyses. TN = trigeminal neuralgia, RS = radiosurgery, DREZ = dorsal root entry zone, RG = retrogasserian, μMLC = micro multi-leaf collimator

Treatment outcome (see Table 1) was measured using the Barrow Neurological Institute (BNI) score for pain and hypaesthesia:

Barrow Neurological Institute (BNI) score

**Table:** 

BNI pain intensity score	
I	No trigeminal pain, no medication
II	Occasional pain, not requiring medication
IIIa	No pain, continued medication
IIIb	Persistent pain, controlled with medication
IV	Some pain, not adequately controlled with medication
V	Severe pain/no pain relief
BNI facial numbness score	
I	No facial numbness
II	Mild facial numbness, not bothersome
III	Facial numbness, somewhat bothersome
IV	Facial numbness, very bothersome

Toxicities were evaluated with Common Toxicity Criteria (CTC) version 3.0.

### Volumetric assessment of the trigeminal nerve and 70 Gy volume determination

Using the Brainlab® iPlan® RT Dose version 4.5.5 (Brainlab® AG, München, Germany) retrospective assessing of treatment volumes as the 70 Gy volume and trigeminal volume inside the 70 Gy volume was done for all patients in Bonn. In order to calculate the trigeminal volume inside the 70 Gy volume, the 70 Gy volume was subtracted from the trigeminal volume and the difference between original trigeminal volume and subtracted trigeminal volume was calculated. The Brainlab® BrainSCAN version 5.31 software in Erfurt had been used for all treated patients from 2001 to 2012. This version of software was able to show trigeminal volume inside 70 Gy volume directly at the dose-volume histograms. From 2012 all treatments in Erfurt had been planned with Cyberknife® software MultiPlan® version 4.6 (Accuray Inc., Sunnivale, USA), which also calculates trigeminal volume inside the 70 Gy volume directly.

### Statistical methods

Statistical univariate and multivariate analysis was performed using IBM® SPSS Statistic 20® (IBM®, Armonk, NY, USA) software to determine which patient and treatment related factors influenced the outcome (BNI score for pain and hypaesthesia). Factors evaluated included patient’s age and sex, TN form (classic = type 1, non classic = type 2: MS-related, symptomatic, atypical), FU time, fixation (framebased/frameless), collimation (4 mm/5 mm cones/μMLC), target point (DREZ/RG), and trigeminal 70 Gy volume, respectively. For numerical data t-tests and for categorical data Fisher’s exact test and Pearson χ^2^ Test were used. Statistical significance was assessed as *p* < 0.05.

## Results

Two patients did not have sufficient FU; for the patients with an available FU (*n* = 53), the mean FU was 30.7 months, range 2–200 months (Table 1).

The overall rate of treatment success, defined as BNI pain I-IIIa (=painfree w or w/o medication) was 69%, the overall rate for painfree w/o medication (= BNI I-II) was 29%.

In the patients with classic TN (type 1), the BNI I-IIIa rate was 91.6% at first FU (33/36, 1 lost FU) and 88.8% at last available FU (32/36, 1 lost FU) and the BNI I-II rate was 47.2% at first FU (17/36, 1 lost FU) and 38.8% (14/36, 1 lost FU) at last available FU.

**Fig. 4:**
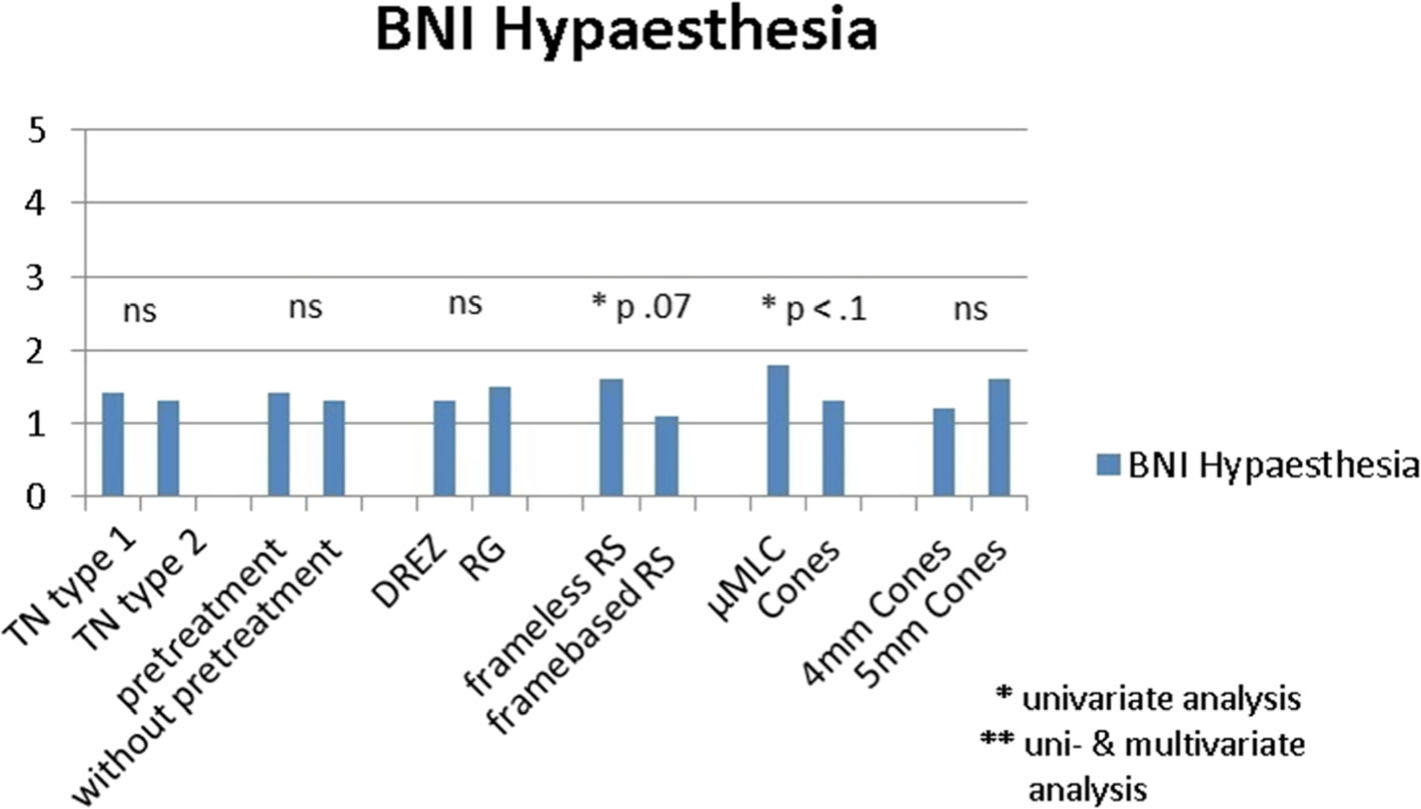
BNI hypaesthesia scores for different subgroups. Results of univariate analyses. TN = trigeminal neuralgia, RS = radiosurgery, DREZ = dorsal root entry zone, RG = retrogasserian, μMLC = micro multi-leaf collimator

The mean BNI score was not significantly different between the classic (type 1) and the non classic (type 2) TN (2.2 vs. 2.8) (Figs. 3 and 4). In the patients with MS-related TN, the BNI I-IIIa rate at first FU was 64.3% (9/14, 1 lost FU) and 50.0% (7/14, 1 lost FU) at last available FU, significantly lower than the rate of classic TN, *p* = 0.03 and *p* = 0.003 respectively. The BNI I-II rate was 21.4% (3/14, 1 lost FU) at first FU and 14.3% (2/14, 1 lost FU) at last available FU, not significantly different to the classic TN. The rate for symptomatic and atypical TN was statistically not meaningful evaluable, since this group was represented by 3 patients, only.

**Fig. 5:**
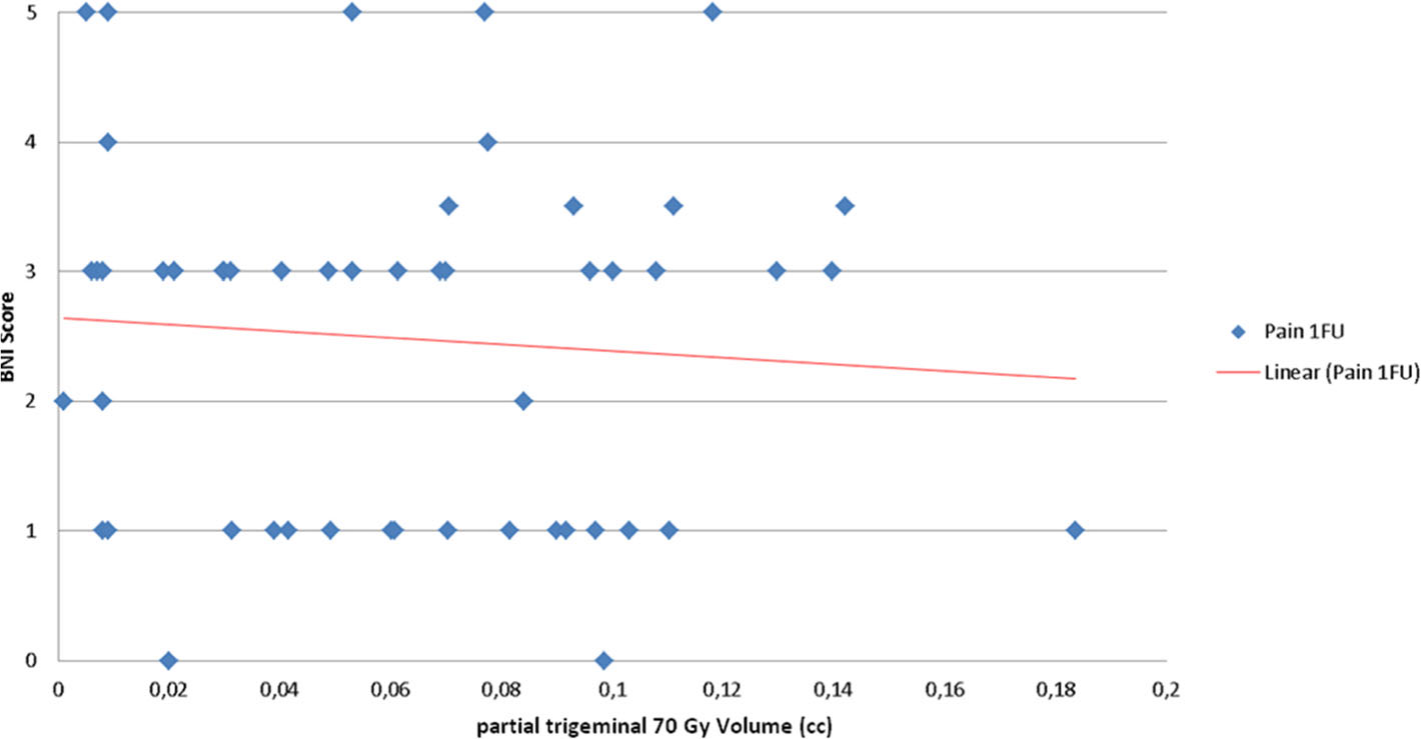
Relation of BNI pain at 1. FU and partial trigeminal 70 Gy volume. BNI=Barrow Neurological Institute, FU = follow-up

We found only a weak statistical tendency of a better outcome in case of framebased SRS vs. frameless robotic SRS for BNI pain (framebased: 2.1 vs. frameless: 2.7, *p* = 0.07) and BNI hypaesthesia at first FU (framebased: 1.1 vs. frameless: 1.6, *p* = 0.07) (Figs. 3 and 4).

No statistical difference between 4 mm and 5 mm cones but a statistical tendency for a better outcome was found when using cones instead of μMLC. BNI pain score: cones: 2.2 vs. μMLC: 3.2; *p* = 0.056; BNI hypaesthesia score: cones: 1.3 vs. μMLC: 1.8; *p* = 0.098 (Figs. 3 and 4).

Further on, we found a significant difference of the BNI pain score at 1st FU depending to the target point (Figs. 3 and 4), the DREZ target proofed to be better than the RG target (2.1 vs. 3.1, *p* = 0.01), but there were no significant differences for the BNI I-IIIa and BNI I-II rates at the first follow up.

The overall rate of new hypaesthesia BNI II-III was 9.4% (*n* = 5, 2 lost FU) and only in *n* = 1 a new BNI hypaesthesia IV was noted. This female patient with a classic TN had at the same time a BNI pain outcome score of I (no trigeminal pain attacks). In one patient the hypaesthesia BNI IV was pre-existing.

In patients with classical TN, the new hypaesthesia BNI II-III rate was 13.8% (5/36, 1 lost FU), in patients with MS-related TN the BNI II-III hypaesthesia rate was 7.1% (4/14, 1 lost FU), this was not significantly different. Again, the rate for symptomatic and atypical TN was statistically not meaningful evaluable, since this group was represented by 3 patients, only.

Concerning to possible (CTC) toxicities no patient with a decreased corneal sensation (dry eye syndrome) was found. None of the patients reported jaw (masseter) weakness or anaesthesia dolorosa. There were no cases of morbidity in areas other than the trigeminal nerve, such as any brainstem injury.

### Multivariate analysis

The univariate statistical results were further analyzed with multivariate analysis. The multivariate analysis of potential influencing factors showed no significant difference in terms of outcome (BNI pain and BNI hypaesthesia scores) for age, gender, MS or not MS-related, w or w/o pretreatment, framebased or frameless SRS.

The target point remains the only significant variable in the multivariate analysis for the BNI pain score at 1st FU, the DREZ target proofed to be better than the RG target. No persistent significant variable could be found in the multivariate analysis regarding the BNI hypaesthesia score (Figs. 3 and 4).

### Influence of the partial trigeminal volume inside the 70 Gy isodose sphere

The percentage of the volume of the trigeminal nerve inside the approximately spherical 70 Gy volume (=partial trigeminal volume) ranged from 10 to 50% (mean: 36.1%, median: 34.6%). In absolute terms this means that the trigeminal volume irradiated with a minimal dose of 70 Gy ranged from 0.001 to 0.05 cc (mean: 0.020 cc, median: 0.018 cc), when excluding the one case with a prescription dose of 70 Gy, it ranged from 0.004 to 0.05 (mean: 0.022 cc, median: 0.020 cc).

When dichotomizing to a partial trigeminal TN volume of 40% and when comparing the cases with ≤40% partial volume vs. > 40% partial volume a statistical significance for a higher grade of BNI hypaesthesia (*p* = 0.01) and a statistical tendency of a lower grade of BNI pain at 1st FU (*p* = 0.07) were observed, meaning a larger partial trigeminal volume correlated weakly with a better outcome for the BNI pain score (Fig. 5), but at the same time correlated significantly with a higher score of BNI hypaesthesia.

## Discussion

An acceptable effectiveness (overall BNI I-IIIa success rate of 69% and of 88% in the classic TN subgroup) with low side effects (< 11%) was found in our 2 center series of LINAC SRS for TN with a total of 55 patients including pretreated and MS-related TN subtypes. In addition, our series showed that a SRS target closer to the brainstem (DREZ target) and tendencially also a larger target volume (more precisely: a larger partial trigeminal 70 Gy volume) were associated with an improved responsiveness without necessarily provoking more relevant side effects. No significant relationship was found between age, gender, presence of MS diagnosis, surgical or ablative pre-treatment and SRS outcome.

Radiosurgery is no curative therapy, but a valuable treatment option for TN [29]. It is to be noted that in Europe and in Germany the option SRS is used comparatively rarely in TN. Although this is also seen worldwide, the frequency of its application is once again reduced by a factor of 5–10 in Europe [30]. The literature on SRS in TN is traditionally dominated by GK series [31], the largest series of patients with the longest follow-up can be found there [16, 32].

One of the most experienced single GK centers in Pittsburgh reported a success rate of 85% (complete pain free 70%) in 220 patients with a mean FU of 2 years [15]. Other GK series with a longer FU reported a decrease of pain relief of > 25% after 5 years [24, 31, 33, 34].

Only few studies [3, 9, 33] have compared different methods of non-drug treatment of TN, including a recent study that compared the standard (micro-)neurosurgical therapy (microsurgical vascular decompression = MVD or Janetta procedure) with GK SRS [34]. This prospective study reported a initial success (painfree = BNI I) rate of 95% vs. 75%, respectively, and a persistent effect after 5 years of 65% vs. 50%, respectively, and after 10 years of 45 vs. 35%, respectively [34]. Although the Janetta decompression had an initial higher success rate than SRS, both therapies showed a considerable loss of success (of 40–50%) after 10 years.

The reported patient numbers in series using LINAC SRS for the treatment of TN are comparatively fewer [19–24, 28, 35–38], consequentially fewer numbers of patients can be reviewed in total, but there are also some smaller prospective LINAC studies (for review see [25]). Synoptically, the reported success rates are very similar to the published rates of GK therapy. However, the rates of treatment-related side effects (hypaesthesia) appear to be significantly more variable (7.5–52%) [25]. This may be due to the fact that the treatment modalities of LINAC SRS are more variable because different LINAC systems with different skull fixations, different monitoring during the irradiation and also different planning and dosage concepts were applied. Naturally, here the GK therapy has the advantage of system homogeneity. Therefore, it was rational at the start to compare different dosing and target concepts for the GK treatment [18, 39, 40]. Regarding the GK therapy as the golden standard, these concepts were essentially transferred to LINAC SRS and modified according to the LINAC system used, which did not always work satisfactorily. There are comparatively only few LINAC studies that have compared intrinsically different treatment modalities with the same LINAC system. One of the most experienced LINAC groups could prove that a dose concept with 90 Gy was more successful than a dose concept with a maximum dose of 70 Gy [41, 42]. In our series, we therefore consistently used 90 Gy as prescribed dose in the isocenter with only one exception in a previously irradiated patient, where we used 70 Gy instead.

We consider our series as a viable contribution to the LINAC radiosurgery literature for treatment of TN. The relatively long observation period reflects one of the strengths of our study. Of importance, we have used a very homogeneous dosing concept and a similar 6 MV LINAC system with an almost identical collimation using 4–5 mm μMLC or cones over the past 15 years, so that we are able to report about a relatively homogeneous patient collective. Our series included framebased and advanced robot-assisted approaches and our cohort consisted of two proportionally comparable groups of patients. In addition, we also have proportionally comparable groups with an emphasis to the classic target points, the DREZ and the RG target, so we could compare these too in terms of outcome and side effects. As the result, there was no significant difference in the multivariate analysis between framebased and frameless robotic SRS, here we found only a weak statistical tendency for a slightly better outcome (BNI pain and BNI hypaesthesia) for the framebased SRS in the univariate analysis. We found a significant difference of BNI pain (1st FU or last FU) when comparing the two used target points, the DREZ target proved to be better than the RG target.

Comparable to a study on GK SRS [43], we can here confirm that the brainstem nearer target promises a higher success rate than the more brainstem distant target. But it has to be noted that another GK study did not confirm that result [44]. Hence, both targets may be considered from our point of view. The RG target still has its justification in individual cases, as it can be very well verified, both during planning and during irradiation in case of image-guided frameless SRS, because of the closer bony relation and it provides a safe target alternative especially for individual cases with an anatomically narrow prepontine space, in particular of relevance in microsurgical pretreated cases, if through scarred displacements of the brainstem the prepontine space is reduced (Fig. 2).

With the volumetric evaluation of the partial trigeminal volume within the 70 Gy isodose, we gained an additional parameter that was statistically significant in our patient series. The 70 Gy isodose threshold for a sufficient effective analgesic isodose was empirically selected after literature review of both, the relevant GK literature and the LINAC literature [18, 42]. If one postulates that the 70 Gy volume remains relatively the same because of a very similar system configuration for each patient in our series, then the individual diameter of the nerve and the individual centering of the approximately spherical 70 Gy volume will have the most influence on the percentage and absolute value of the partial trigeminal volume within this 70 Gy sphere. Our hypothesis that the volume fraction of the trigeminal nerve within this postulated minimal effective threshold may play a role for the analgetic impact of LINAC SRS has been confirmed in our series to the extent that we observed a statistical tendency of an improved outcome for BNI pain in case of a larger partial 70 Gy volume (when performing a dichotomy at the 40% partial 70 Gy volume). As a consequence, the planning should focus on a perfect centering and a sufficient diameter of the nerve to increase the proportion of the nerve which receives a 70 Gy minimum dose. But otherwise and with it we see also significantly more BNI hypaesthesia, meaning the more trigeminal tissue is captured by the 70 Gy isodose the better the response to pain but the more new radiogenic hypaesthesia has to be accepted, however the hypaesthesia rate in our series was still relatively low at about 11%.

We did not look specifically at the length of the nerve inside the treatment volume, but of course the length of the nerve and the volume of the nerve correlate with each other. Flickinger et al. using GK SRS examined the influence of the nerve length in the treatment volume and found a similar connection, viz. the longer the irradiated nerve length was, the more side effects in form of sensory disturbances occurred. However and in contrast to our findings, no difference was found regarding pain outcome [39].

The possible higher rate of hypaesthesia has to be communicated to the patients and ultimately, this should in our opinion be discussed with the patients, whether they accept a higher rate of hypaesthesia as a calculated risk to achieve maybe a better pain relief in LINAC SRS for TN.

### Limitations

The low patient case number is surely a limiting factor. Our mean follow up is not sufficient for long-term results. We prospectively collected our patient series and treatment data, but partly evaluated retrospectively our outcome data. The parameter 70 Gy volume has only been found empirically and may only be valid for our series and cannot be generalized. This applies to all our results, but we hope that our carefully collected data will be nevertheless a significant contribution to future meta-analyzes on LINAC SRS in trigeminal neuralgia.

## Conclusions

LINAC SRS is no curative therapy in TN, but it is a valid treatment option for TN because of its acceptable effectiveness even in pretreated and MS-related cases. A target definition closer to the brainstem side and tendencially also a larger target volume inside the 70 Gy isodose line were correlated with a better pain outcome in our series. Frameless robotic SRS can be performed in the same quality as framebased SRS when using a dedicated LINAC system. Large-scale controlled trials comparing different approaches are required in order to evidently confirm the potential value of LINAC SRS and to promote its wider application.

## Abbreviations

BNI: Barrow Neurological Institute
DREZ: Dorsal root entry zone
DVH: Dose-volume histogram
FU: Follow-up
GK: Gamma Knife
LINAC: Linear accelerator
MS: Multiple sclerosis
MVD: Microsurgical vascular decompression
OAR: Organs at risk
PRR: Percutaneous retrogasserian rhizotomy
RG: Retrogasserian
SRS: Stereotactic radiosurgery
TN: Trigeminal neuralgia
μMLC: Micro multi-leaf collimator
